# Supplementary material for: Long-Term Persistence of Mitochondrial DNA Instability among HCV-Cured People Who Inject Drugs
Source: Biomedicines. 2022 Oct 12;10(10):2541. doi: 10.3390/biomedicines10102541 (PMC9599189; doi:10.3390/biomedicines10102541)
Supplement: Supplementary file 1 [file biomedicines-10-02541-s001.zip › biomedicines-1905790-supplementary.pdf]

Supplementary file: Long-term persistence of mitochondrial DNA instability among HCV-cured people who inject drugs. Durand et al.

Figure S1: Flowchart of the study population

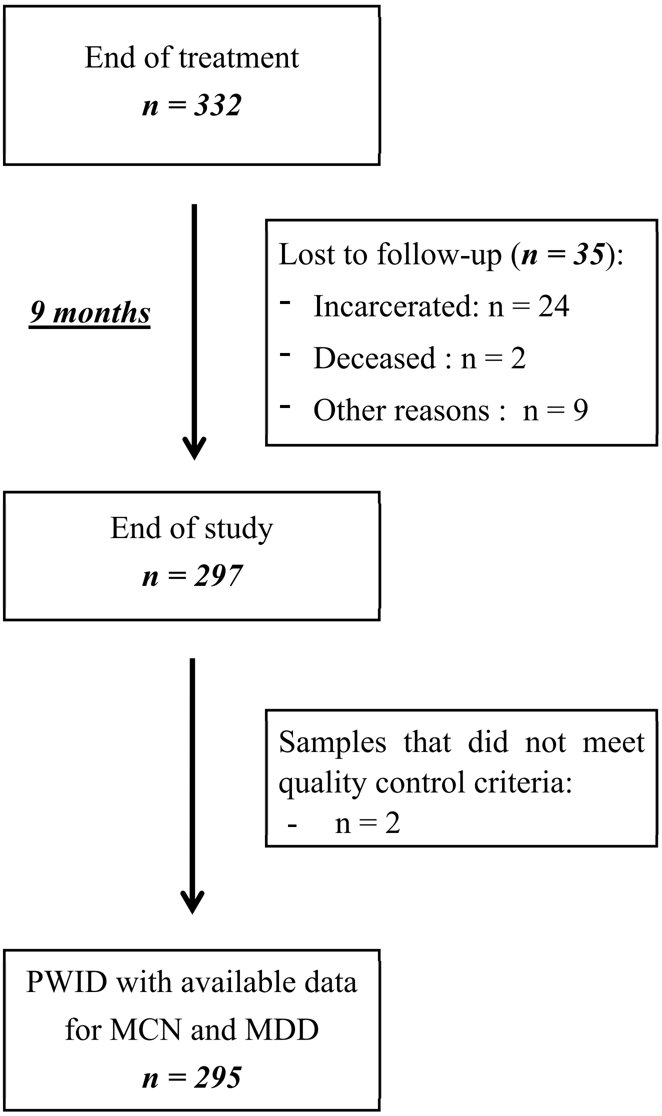

Table S1: Factors associated with high MCN loss.

	n(%)	UNIVARIATE		MULTIVARIATE	
		**RR [95%CI]**	***p-values***	**aRR [95%CI]**	***p-values***
**SOCIO-DEMOGRAPHIC DATA**					
**Age**			0.12		0.15
Under 35 years	37 (12.5)	Ref.		Ref.	
35 to 45 years	164 (55.6)	1.16 [0.70; 1.93]		1.14 [0.69; 1.88]	
45 to 55 years	72 (24.4)	0.68 [0.36; 1.29]		0.68 [0.36; 1.28]	
Over 55 years	22 (7.4)	1.12 [0.54; 2.31]		1.03 [0.48; 2.22]	
**TREATMENTS**					
**Previous exposure to DAA**			0.68		0.83
SOF400/DCV60	149 (50.5)	Ref.		Ref.	
SOF400/DCV90	119 (40.3)	1.14 [0.81; 1.61]		1.10 [0.78; 1.55]	
SOF400/DCV/RBV	27 (9.1)	1.20 [0.69; 2.08]		1.22 [0.69; 2.15]	
**ARV exposure**			0.27		
Not receiving ARV	158 (53.6)	Ref.			
Receiving ARV treatment	137 (46.4)	1.20 [0.87; 1.66]			
**Methadone maintenance therapy**			0.13		0.11
No	36 (12.2)	Ref.		Ref.	
Yes	259 (87.8)	0.71 [0.47; 1.07]		0.69 [0.46; 1.04]	
**DRUG/SUBSTANCE CONSUMPTION**					
**Heroin status at 9 months post-treatment**			0.42		
No use	174 (59.0)	Ref.			
Still injecting	121 (41.0)	0.87 [0.62; 1.22]			
**Methamphetamine status at 9 months post-treatment**			0.43		
No use	262 (88.8)	Ref.			
Still smoking	33 (11.2)	1.21 [0.77; 1.92]			
**Hazardous drinking* at 9 months post-treatment**			0.98		
No	247 (83.7)	Ref.			
Yes	48 (16.3)	1.00 [0.65; 1.55]			

*: *score above 4 for men or 3 for women on the AUDIT-C scale. All statistical tests were non-parametric tests.*

Table S2: Factors associated with high MDD accumulation.

		UNIVARIATE		MULTIVARIATE	
	**n(%)**	**RR [95%CI]**	***p-values***	**aRR [95%CI]**	***p-values***
**SOCIO-DEMOGRAPHIC DATA**					
**Age**			0.31		0.28
Under 35 years	37 (12.5)	Ref.		Ref.	
35 to 45 years	164 (55.6)	0.70 [0.46; 1.09]		0.71 [0.45; 1.11]	
45 to 55 years	72 (24.4)	0.83 [0.52; 1.35]		0.81 [0.50; 1.32]	
Over 55 years	22 (7.4)	0.52 [0.22; 1.23]		0.49 [0.21; 1.15]	
**TREATMENTS**					
**Previous exposure to DAA**			0.36		0.32
SOF400/DCV60	149 (50.5)	Ref.		Ref.	
SOF400/DCV90	119 (40.3)	0.82 [0.57; 1.17]		0.84 [0.58 ; 1.20]	
SOF400/DCV/RBV	27 (9.1)	1.17 [0.70; 1.93]		1.27 [0.76 ; 2.12]	
**ARV exposure**			0.21		
Not receiving ARV	158 (53.6)	Ref.			
Receiving ARV treatment	137 (46.4)	0.81 [0.58 ; 1.13]			
**Methadone maintenance therapy**			0.66		
No	36 (12.2)	Ref.			
Yes	259 (87.8)	0.90 [0.56; 1.43]			
**DRUG/SUBSTANCE CONSUMPTION**					
**Heroin status at 9 months post-treatment**			0.76		
No use	174 (59.0)	Ref.			
Still injecting	121 (41.0)	1.05 [0.76; 1.46]			
**Methamphetamine status at 9 months post-treatment**			0.22		
No use	262 (88.8)	Ref.			
Still smoking	33 (11.2)	1.34 [0.87; 2.07]			
**Hazardous drinking* at 9 months post-treatment**			0.29		
No	247 (83.7)	Ref.			
Yes	48 (16.3)	1.23 [0.84; 1.86]			

***:** *score above 4 for men or 3 for women on the AUDIT-C scale. All statistical tests were non-parametric tests.*
